# Neurturin overexpression in dopaminergic neurons induces presynaptic and postsynaptic structural changes in rats with chronic 6-hydroxydopamine lesion

**DOI:** 10.1371/journal.pone.0188239

**Published:** 2017-11-27

**Authors:** David Reyes-Corona, Nallely Vázquez-Hernández, Lourdes Escobedo, Carlos E. Orozco-Barrios, Jose Ayala-Davila, Mario Gil Moreno, Miriam E. Amaro-Lara, Yazmin M. Flores-Martinez, Armando J. Espadas-Alvarez, Manuel A. Fernandez-Parrilla, Juan A. Gonzalez-Barrios, ME Gutierrez-Castillo, Ignacio González-Burgos, Daniel Martinez-Fong

**Affiliations:** 1 Departamento de Fisiología, Biofísica y Neurociencias, Centro de Investigación y de Estudios Avanzados, Ciudad de México, México; 2 Laboratorio de Psicobiología, División de Neurociencias, Centro de Investigación Biomédica de Occidente, IMSS, Guadalajara, Jalisco, México; 3 CONACYT—Medical Research Unit in Neurological Diseases, National Medical Center "Siglo XXI", IMSS, Mexico City, Mexico; 4 Laboratorio de Neurobiología del Apetito, Departamento de Farmacología, Centro de Investigación y de Estudios Avanzados, Ciudad de México, México; 5 Laboratorio de Medicina Genómica, Hospital Regional 1º de Octubre, ISSSTE, Ciudad de México, México; 6 Departamento de Biociencias e Ingeniería, Centro Interdisciplinario de Investigaciones y Estudios sobre Medio Ambiente y Desarrollo, Instituto Politécnico Nacional, Ciudad de México, México; 7 Programa de Doctorado en Nanociencias y Nanotecnología, Centro de Investigación y de Estudios Avanzados, Ciudad de México, México; Thomas Jefferson University, UNITED STATES

## Abstract

The structural effect of neurturin (NRTN) on the nigrostriatal dopaminergic system in animals remains unknown, although NRTN has been shown to be effective in Parkinson’s disease animal models. Herein, we aimed to demonstrate that NRTN overexpression in dopaminergic neurons stimulates both neurite outgrowths in the nigrostriatal pathway and striatal dendritic spines in aging rats with chronic 6-hydroxydopamine (6-OHDA) lesion. At week 12 after lesion, pTracer-mNRTN-His or pGreenLantern-1 plasmids were intranigrally transfected using the NTS-polyplex nanoparticles system. We showed that the transgenic expression in dopaminergic neurons remained until the end of the study (12 weeks). Only animals expressing NRTN-His showed recovery of tyrosine hydroxylase (TH)+ cells (28 ± 2%), their neurites (32 ± 2%) and the neuron-specific cytoskeletal marker β-III-tubulin in the substantia nigra; striatal TH(+) fibers were also recovered (52 ± 3%), when compared to the healthy condition. Neurotensin receptor type 1 levels were also significantly recovered in the substantia nigra and striatum. Dopamine recovery was 70 ± 4% in the striatum and complete in the substantia nigra. The number of dendritic spines of striatal medium spiny neurons was also significantly increased, but the recovery was not complete. Drug-activated circling behavior decreased by 73 ± 2% (methamphetamine) and 89 ± 1% (apomorphine). Similar decrease was observed in the spontaneous motor behavior. Our results demonstrate that NRTN causes presynaptic and postsynaptic restoration of the nigrostriatal dopaminergic system after a 6-OHDA-induced chronic lesion. However, those improvements did not reach the healthy condition, suggesting that NRTN exerts lesser neurotrophic effects than other neurotrophic approaches.

## Introduction

Neurturin (NRTN) together with glial cell line-derived neurotrophic factor (GDNF), persephin and artemin constitute the GDNF family ligands (GFL) [1, 2]. Those neurotrophic factors bind high-affinity receptors collectively known as GDNF family receptors (GFRas), which share structural and functional likenesses [3]. As those receptors lack an intracellular domain, they are anchored to cell membrane by a glycosylphosphatidylinositol (GPI) residue and activate intracellular signaling pathways through the proto-oncogene RET [3]. NRTN binds to GFRα2 with high affinity, but can also bind to GFRα1 to activate RET [4, 5] and promote survival, differentiation, and maintenance in many neuronal populations, including dopaminergic neurons [6].

The neurotrophic role of NRTN in dopaminergic neurons of the substantia nigra has been less explored than that of GDNF during the ontogeny and adulthood of dopaminergic neurons. Most of the studies on NRTN in these fields have been performed in comparison with GDNF. Although their neurotrophic effects are similar on dopaminergic neurons, NRTN seems to act afterward GDNF as suggested by the differential expression pattern of these neurotrophic factors during postnatal development of the rat mesencephalon [6–8]. While GDNF is expressed by parvalbumin-positive GABAergic neurons in the striatum, not in medium spiny neurons [9] or in the midbrain [10], NRTN is expressed in both the substantia nigra and striatum by still unidentified cells [7, 11]. In adulthood, NRTN mRNA levels are twice the levels observed in the striatum, but NRTN protein levels in both nuclei remain as low as other neurotrophic factors, thus contributing only to the maintenance of dopaminergic nigrostriatal system [11].

The effect of NRTN on survival, neuritogenesis and regeneration of dopaminergic neurons in the adult brain is not fully understood. Experiments in ventral mesencephalon cultures or embryonic dopaminergic neurons [7] have shown that NRTN promotes potent survival effect comparable with GDNF [6] and that NRTN exhibits survival-promoting actions on both developing and mature dopaminergic neurons [7]. On the contrary, other studies have shown relatively low expression of NRTN in the striatum and no pattern of developmental regulation in the substantia nigra [11] suggesting that NRTN does not regulate natural cell death in dopaminergic neurons, either as a target-derived or as a local paracrine factor [11]. In addition, studies on developing dopaminergic neurons *in vitro* have shown that NRTN lacks the neuritogenic and hypertrophic activity of GDNF [6]. Furthermore, studies on knockout of NRTN or its receptor GFRα2 in mice have reported normal number of dopaminergic neurons showing that endogenous NRTN is not the survival factor for these neurons [12, 13], although those animals exhibit abnormalities in enteric, parasympathetic and sensory neurons [12, 14, 15]. Since a significant reduction in GFRα2 also occurs in NRTN knockout mice and given the conserved number of dopaminergic neurons in NRTN or GFRα2 knockout mice [12, 13], it is plausible to suggest that GDNF can substitute for NRTN [12]. This suggestion is supported by the finding that the absence of RET, the signal-transducing receptor for GFRα1 and GFRα2 [16], causes progressive and late degeneration of nigral dopaminergic neurons [17]. In contrast, the neuritogenic effect of NRTN is well documented in developing and adult central noradrenergic neurons [18].

To date, preventive and restorative experiments in animal models of Parkinson’s disease (PD) [19, 20] and clinical trials [21] have been focused in demonstrating the recovery of enzymatic (tyrosine hydroxylase; TH) phenotype and normal motor behavior. All these studies *in vivo* only suggest that NRTN might stimulate axonal elongation and dendritic branching outgrow in the dopaminergic nigrostriatal system. However, experimental evidence has not yet provided on this issue.

The 6-hydroxydopamine (6-OHDA) lesion model in the rat has proven to be useful to explore the neurotrophic effect of NRTN [22–25]. An advantage of the striatal 6-OHDA lesion is that the time course of apoptotic death of nigral dopaminergic neurons is well characterized, lasting 4 weeks after injection with a maximum peak between days 3 and 21 after the lesion [26]. In this lesion model, the maximum decrease in the phenotype markers (TH and β-III-tubulin) is reached at week 4 after injury and 10–20% of TH(+) cells remain unaltered along the whole life of the rat [26]. Therefore, we used this striatal lesion model to explore whether the overexpression of NRTN in nigral dopaminergic neurons of rats with advanced lesion can cause presynaptic and postsynaptic restoration of the nigrostriatal dopaminergic system. To test this hypothesis, we used the neurotensin (NTS)-polyplex nanoparticles (NPs) system for NRTN gene delivery because of its proven ability to transfect dopaminergic neurons of the substantia nigra *in vitro* [27] and *in vivo* [28–31]. NTS-polyplex NPs exploits the highly enriched expression of NTS receptor type 1 (NTSR1) in the plasma membrane of dopaminergic neurons to transfer genes into these neurons via internalization of NTSR1 [27, 32–34]. Thus, transgene expression and structural, molecular, biochemical and behavioral variables were assessed over time until week 12 after transfection. Our results demonstrate that NRTN overexpression in dopaminergic neurons induces regeneration of the nigrostriatal system and neuronal plasticity in the striatum of rats with chronic 6-OHDA lesion.

## Materials and methods

### Plasmids

pTracer-mNRTN-His (6,530 bp) coding for mouse NRTN (GenBank, NM_008738.2) in fusion with V5 and His Tag under the control of human elongation factor 1-α promoter (hEF1-α) was used. Briefly, a 587 bp fragment of the coding sequence for mNRTN encompassed in the plasmid pcDNA3-mNRTN (kindly donated by Dr. Mart Saarma and Dr. Runeberg-Roos; Institute of Biotechnology, University of Helsinki, Helsinki, Finland) was amplified through PCR by eliminating the stop codon. Then, the amplified fragment was cloned between the EcoRI restriction sites of pTracer^TM^-EF/V5-His A (Invitrogen Corp; Carlsbad, CA, USA), which also encompasses the coding sequence for green fluorescent protein (GFP) under the control of cytomegalovirus (CMV) promoter. Full NRTN sequence and correct Tag addition were confirmed by automatic sequencing (BigDye v3.1; Applied Biosystems; Foster City, CA, USA).

pGreenLantern-1 (5,030 bp) coding for GFP under the control of CMV promoter (Gibco BRL; Grand Island, NY, USA) was used as a negative control.

### Assembly of NTS-polyplex NPs

The conjugation of poly-L-lysine with NTS and a fusogenic peptide (NTS-FP-PLL) is described in detail elsewhere [34–36]. Briefly, NTS (Sigma; Saint Louis, MO, USA) and FP (GLFEAIAEFIEGGWEGLIEGCAKKK; purity >90%; RS Synthesis; Louisville, KY, USA) was cross-linked with poly-L-lysine (48 kDa mean molecular mass; Sigma-Aldrich; Saint Louis, MO, USA) using succinimidyl 3-(2-pyridyldithio)propionate (SPDP; Thermo Scientific pierce; Rockford, IL, USA). Fast performance liquid chromatography was used to purify the SPDP-derivatives and the NTS-SPDP-(FP-SPDP)-poly-L-lysine conjugate, the NTS carrier. NTS-polyplex NPs were assembled by electrostatically binding the karyophilic peptide (KP; MAPTKRKGSCPGAAPNKPK; 90% purity; RS Synthesis; Louisville, KY, USA) and the NTS carrier to a plasmid DNA (pDNA) [34–36]. The criterion of retardation and retention assays [27, 33–35] was used to calculate the optimum molar ratio for NTS-polyplex NPs components, which was 30 nM of pDNA: 30 μM KP: 810 nM NTS carrier for the two plasmids. At this molar ratio, the concentration of NTS used was 385.6 pmol/μL, calculated by measurements of ^125^I-NTS [35, 37–39]. The concentrations of pDNAs were 129.01 ng/μL of pTracer-mNRTN-His and 99.59 ng/μL of pGreenLantern-1.

### Animals

Male Wistar rats weighing 220 ± 10 g at the beginning of experiments were used. The protocol (Permit Number: 162–15) was approved by the Internal Committee for the Care and Use of Laboratory Animals of the Center for Research and Advanced Studies of the National Polytechnic Institute (CINVESTAV-IPN) in accordance with the current Mexican legislation, NOM-062-ZOO-1999 and NOM-087-ECOL-1995 (Secretaría de Agricultura, Ganadería, Desarrollo Rural, Pesca y Alimentación; SAGARPA). All efforts were made to minimize animals suffering and the number of animals used was kept to a minimum by the experimental design.

### Intracranial injections

Rats were anaesthetized with ketamine/xylazine (120 / 9 mg/kg; i.p.) and placed on a stereotaxic frame (Stoelting; Wood Dale, ILL, USA). A single dose of 6-OHDA solution (20 μg free base in 3 μL of phosphate-buffered saline solution (PBS) containing 0.2% ascorbic acid; Sigma-Aldrich; St. Louis, MO, USA) was injected into the left striatum at the coordinates: AP, +7.7 mm from the interaural midpoint; ML, +4.0 mm from the intraparietal suture; DV, -5.4 mm from the dura mater [26, 28, 29, 38]. The flow rate of injection was 0.1 μL/min [26]. NTS-polyplex NPs (1.5 μL) were injected into the left substantia nigra of rats (440 ± 15 g of body weight) with advanced (12 weeks) 6-OHDA lesion at the coordinates: AP, +2.7 mm from the interaural midpoint; ML, +2.2 mm from the intraparietal suture; DV,– 6.8 mm from the dura mater. The flow rate of injection was 0.1 μL/min.

### Circling behavior test

Ipsilateral or contralateral turning behaviors were induced consecutively (1 day after another) by R-apomorphine sulfate (0.5 mg/kg of body weight; i.p.) and methamphetamine (8 mg/ kg of body weight; i.p.). Drug-induced turning behavior was recorded at 1-min intervals over 40 min (apomorphine) or 180 min (methamphetamine) as described previously [28, 31, 40].

### Limb-use asymmetry (“cylinder”) test

The control and transfected rats were been placed in a transparent acrylic cylinder (30 cm tall, 20 cm diameter) and the first 20 contacts made with the ipsilateral, the contralateral and both (simultaneously) paws were video recorded. The time required to generate this number of observations varied from animal to animal. The asymmetry score was calculated as the number of “ipsi” observations plus ½ the number of “both” observations, divided by the total number of observations (ipsi plus contra plus both), and the quotient multiplied by 100 [41]. The test was been performed without conditioning prior to recording, between 10:00 h and 12:00 h.

### Vibrissae-evoked forelimb placing test

The left and right vibrissae of every rat were brushed against the edge of a tabletop to evoke the forelimb placing response. The animal was held in midair with all limbs hanging freely so that no weight was supported by any of the limbs or the tail. The normal motor response elicited is a quick, accurate reaching-type movement of the forelimb ipsilateral to the stimulated vibrissae that quickly terminates when the ventral surface of the paw lands on the tabletop. Results of placing forelimb on the table surface in response to vibrissae stimulation were scored as percent of successful placing out of ten trials for each limb (ipsilateral or contralateral) for each rat, in trials scored as “0”, the limb does not move [42]. The test was been performed without previous conditioning between 11:00 h and 13:00 h.

### Reverse transcription-polymerase chain reaction (RT-PCR)

Total RNA was isolated from fresh substantia nigra by homogenization in 1 mL of Trizol (Invitrogen Corporation; Carlsbad, CA, USA) [28, 31, 43]. Total RNA (5 μg), previously treated with RNase-free DNase, was transcribed using SuperScript II reverse transcriptase (200 U) and 0.1 mg of oligo dT (Invitrogen Corporation; Carlsbad, CA, USA). One μL of the reverse transcribed product was amplified in a thermocycler (Gene Amp PCR System 9700; Applied Biosystems; Foster City, CA, USA) using 1 nmol of each sense and antisense primers and 1 U of Platinum Taq DNA polymerase (Invitrogen Life Technologies; San Diego, CA, USA) in a final volume of 50 μL. To amplify a 176 bp fragment of NRTN-His (GenBank, NM_008738.2), the primers were 5´-GCCTATGAGGACGAGGTGTC-3´ (forward) and 5´-AGACCGAGGAGAGGGTTAGG-3´ (reverse). To amplify a 199 bp fragment of GFP (GenBank, AF188479), the primers were 5´-TACAAGACGCGTGCTGAAGT-3´ (forward) and 5´-CAATGTTGTGGCGAATTTTG-3´ (reverse). A 217 bp fragment of β-actin (GenBank, NM_031144.3) was amplified as internal control using the primers 5´-CGTAAAGACCTCTATGCCAA-3´ (forward) and 5´ACTCCTGCTTGCTGATCCAC-3´ (reverse). PCR conditions were as follows, an initial denaturation at 94°C for 5 min and 40 cycles of amplification for NRTN-His and 35 cycles for GFP and β-actin. Each cycle consisted of denaturalization (94°C, 30 s), annealing (50°C for NRTN-His, 55°C for GFP and 60°C for β-actin, for 30 s), extension (72°C for 30 s), and additional extension (72°C for 7 min). PCR products were electrophoresed in a 1.5% agarose gel, stained with ethidium bromide and photographed with a UVP BioDoc-It 220 Imaging System (Ultra-Violet Products Ltd; Upland, CA, USA).

### Immunostaining

Rats were perfused through the ascending aorta with 150 mL of cold PBS, followed by 150 mL of 4% paraformaldehyde in PBS. The brains processed as described previously [26, 28, 31, 40]. The immunofluorescence assays were made in 35-μm slices using the following primary antibodies, (1) polyclonal goat anti-TH (1:500; Abcam; Cambridge, MA, USA), (2) polyclonal rabbit anti-GFP (1:500; Chemicon; Temecula, CA, USA), (3) monoclonal mouse anti-His (1:250; Invitrogen Molecular Probes; Eugene, Oregon, USA), (4) polyclonal rabbit anti-β-III-tubulin (1:300; Sigma-Aldrich; St. Louis, MO, USA), (5) monoclonal mouse anti-TH (1:1000; Sigma-Aldrich; St. Louis, MO, USA), (6) polyclonal goat anti-NTSR1 (1:50; Santa Cruz Biotechnology Inc; Dallas TX, USA). The secondary antibodies were, (1) Alexa-350 donkey anti-goat (1:300; Invitrogen Corporation; Carlsbad, CA, USA) or (2) donkey anti-goat aminomethylcoumarin acetate (1:60; AMCA; Jackson ImmunoResearch; Palo Alto, CA, USA), (3) FITC donkey anti-rabbit IgG (1:60; Jackson ImmunoResearch; Palo Alto, CA, USA), (4) Texas-Red horse anti-mouse IgG (1:100; Vector Labs; Burlingame, CA, USA), (5) Texas-Red goat anti-rabbit (1:100; Vector Labs; Burlingame, CA, USA), (6) Alexa 488 chicken anti-mouse (1:200; Invitrogen Molecular Probes; Eugene, Oregon, USA). (7) Alexa 488 donkey anti-goat (1:400; Invitrogen Molecular Probes, Eugene, OR, USA). Finally, the slices were washed with PBS and mounted on glass slides using Vectashield (Vector Laboratories; Burlingame, CA, USA).

The immunohistochemistry staining was carried out on free-floating sections previously washed in PBS and incubated in 1% of H_2_O_2_ for 30 min at room temperature (RT) [28, 30, 40]. The primary antibody was a mouse monoclonal anti-TH antibody (1:1000; Chemicon, Temecula, CA, USA), incubated at 4°C for 24 h. The secondary antibody was a biotinylated anti-mouse IgG (1:200; Vector Labs; Burlingame, CA, USA), incubated at RT for 2 h. The color was developed using ABC kit and 3´3–diaminobenzidine (DAB) according to the manufacturer’s instructions (Vector Laboratory; Burlingame, CA, USA). Sections were mounted on glass slides using Entellan (Merck KGaA; Darmstadt, Germany).

A Leica DMIRE2 microscope (Leica; Nussloch, Germany) was used to observe the immunofluorescence through the filters A for Alexa-350 and AMCA, K3 for FITC and Alexa 488, and TX2 for Texas-Red. ImageJ software v.1.46r (National Institutes of Health; Bethesda, MD) was used to determine the immunofluorescence area density (IFAD) for NTSR1 and TH in the double fluorescence assays. The immunohistochemical staining was analyzed in bright field. The images were digitized with a Leica DC300F camera (Leica Microsystems; Nussloch, Germany). Negative controls were obtained by omitting the primary antibody and replacing it by an irrelevant antibody of the same IgG subclass or using the contralateral substantia nigra without transfection or transfection with pGreenLantern-1.

### Densitometry and neuron counting

ImageJ software v.1.46r (National Institutes of Health; Bethesda, MD) was used to determine the total density area of TH(+) fibers in the substantia nigra and striatum, and to count TH(+) neurons in the substantia nigra [28–30]. All background intensity was eliminated from the immunohistochemical stained area to quantify only TH(+) immunoreactivity. The mean intensity of TH(+) fibers and TH(+) cells were determined at least in 5 anatomic levels along the substantia nigra and striatum (*n* = 3 rats for every experimental condition).

### Dopamine quantification by HPLC

Dopamine content was determined in supernatants from homogenates in 0.1M HClO_4_ of the substantia nigra (1:5) or striatum (1:10) using reversed phase high-performance liquid chromatography (RP-HPLC) and electrochemical detection, as described elsewhere [28, 31, 44, 45]. Five μL of filtrated supernatants were injected into a VeloSep RP-18 reverse-phase column (3 μm, 100 x 3.2 mm; PerkinElmer; Waltham, MA, USA) heated at 30.5°C. The mobile phase buffer was 25 mM NaH_2_PO_4_, 50 mM Na-Citrate, 0.03 mM EDTA, 10 mM diethylamine HCl, 2.2 mM 1-octanesulfonic acid/sodium salt (pH 3.2). One liter of the buffer was mixed with 30 mL of methanol and 22 mL of dimethylacetamide to form the mobile phase that was delivered by a BAS HPLC PM-80- Pump (Bioanalytical Systems; West Lafayette, IN, USA) in isocratic elution mode at 0.5 mL/min. The oxidation potential of the glassy carbon electrode was set by a LC-4C electrochemical detector at +0.75 V with respect to the Ag/AgCl reference electrode (Bioanalytical Systems; West Lafayette, IN, USA). Chromatograms were recorded and analyzed by using ChromGraph 2.34.00 REPORT 2.30 software of Bioanalytical Systems, Inc. The pellets were resuspended in 120 μL of 0.1 M NaOH for protein determination using the Coomassie Plus assay kit (Pierce Biotechnology Rockford; IL, USA) as reported elsewhere [28, 31, 44, 45]. Dopamine content was expressed as pg of DA/μg of protein.

### NRTN-His quantification by enzyme-linked immunosorbent assay (ELISA)

A commercial ELISA kit (# catalogue, AKR-130; Cell Biolabs Inc.; San Diego, CA, USA) was used to measure NRTN-His concentration according to the manufacturer´s instructions. The substantia nigra and striatum were quickly dissected out in cold conditions and homogenized using a tissue pestle grinder (VWR International Ltd; Lutterworth, England) in RIPA buffer containing 1% NP40, 2 mM EDTA, 0.1% SDS, 50 mM Tris-HCl pH 7.4, 0.5% Na-deoxycholate, 150 mM NaCl, 50 mM NaF, 1 mM phenylmethylsulfonyl fluoride and protease inhibitor cocktail (Roche Diagnostics GmbH; Mannhein, Germany). The content of NRTN-His was determined at 450 nm using a microplate absorbance reader with Microplate Manager 6 software (Biorad Laboratories Inc.; Hercules, CA, USA). To normalize the concentration of NRTN-His, protein content in the respective tissue samples was measured using a Pierce Microplate BCA Protein Assay Kit and bovine serum albumin as standard according to manufacturer’s instructions (Thermo Fisher Scientific; Rockford, IL, USA).

### Total intersections and spine density

Six rats per group were used to analyze the dendritic spines of medium spiny neurons (MSNs). These were anesthetized with ketamine (30 mg/kg, i.m.) and sodium pentobarbital (50 mg/kg, i.p.), and they were then perfused with 200 mL of PBS (pH 7.4; 0.01 M) containing sodium heparin (1000 IU/l) as an anticoagulant, and procaine hydrochloride (1 g/L) as a vasodilator [46]. The rats were subsequently perfused with 200 mL of a phosphate-buffered 4% formaldehyde fixative solution. Those solutions were perfused at a rate of 11.5 mL/min. The rat’s brain was then removed and maintained for at least 48 h in 100 mL of fresh fixative solution. The bilateral dorsal striatum was subsequently dissected out and impregnated using a modified version of the Golgi technique [47]. Six MSNs were analyzed per rat in each group (*n* = 6 independent rats in each experimental condition). Dendritic arborization of MSNs was evaluated by using the Sholl analysis [48]. This reflects the complexity of the dendritic arbor by determining the number of intersections of the entire dendrites with concentric circles starting from the center of the cell’s soma. In our analysis, we set the distance between the Sholl circles to 10 μm. In addition, dendritic spines were counted in one 50-μm segment from a primary dendrite of each of the six neurons studied per rat. The density and proportion of thin, mushroom, stubby and wide spines were also determined according to previously established criteria [49–52]. Counting was performed by direct observation at 2,000x using a magnification changer coupled to a light microscope with a 100x APO objective and an image analyzer (LAS 4.0).

### Statistical analysis

All results are expressed as mean ± standard error of the mean (S.E.M.) values, obtained at least from 3 independent experiments (*n* = 3). The difference among the groups was analyzed with repeated-measures one-way ANOVA followed by the Newman-Keuls or Tukey *post hoc* test. Two-way ANOVA and Bonferroni post-test were used to analyze differences among the groups in the quantification of double immunofluorescence assays for NTSR1 and TH. Graph Pad Prism 5.0 software (GraphPad Software Inc.; La Jolla, CA, USA) was used for statistical analysis. Accepted significance was at *P* < 0.05. Dendritic arborization and spine density were analyzed with the one-way ANOVA and Tukey *post hoc* tests. The relative proportional density of each spine type was averaged per rat and the averages were then analyzed per group using the one-way ANOVA and the Bonferroni *post hoc* tests.

## Results

### NRTN-His expression

**Fig 1:**
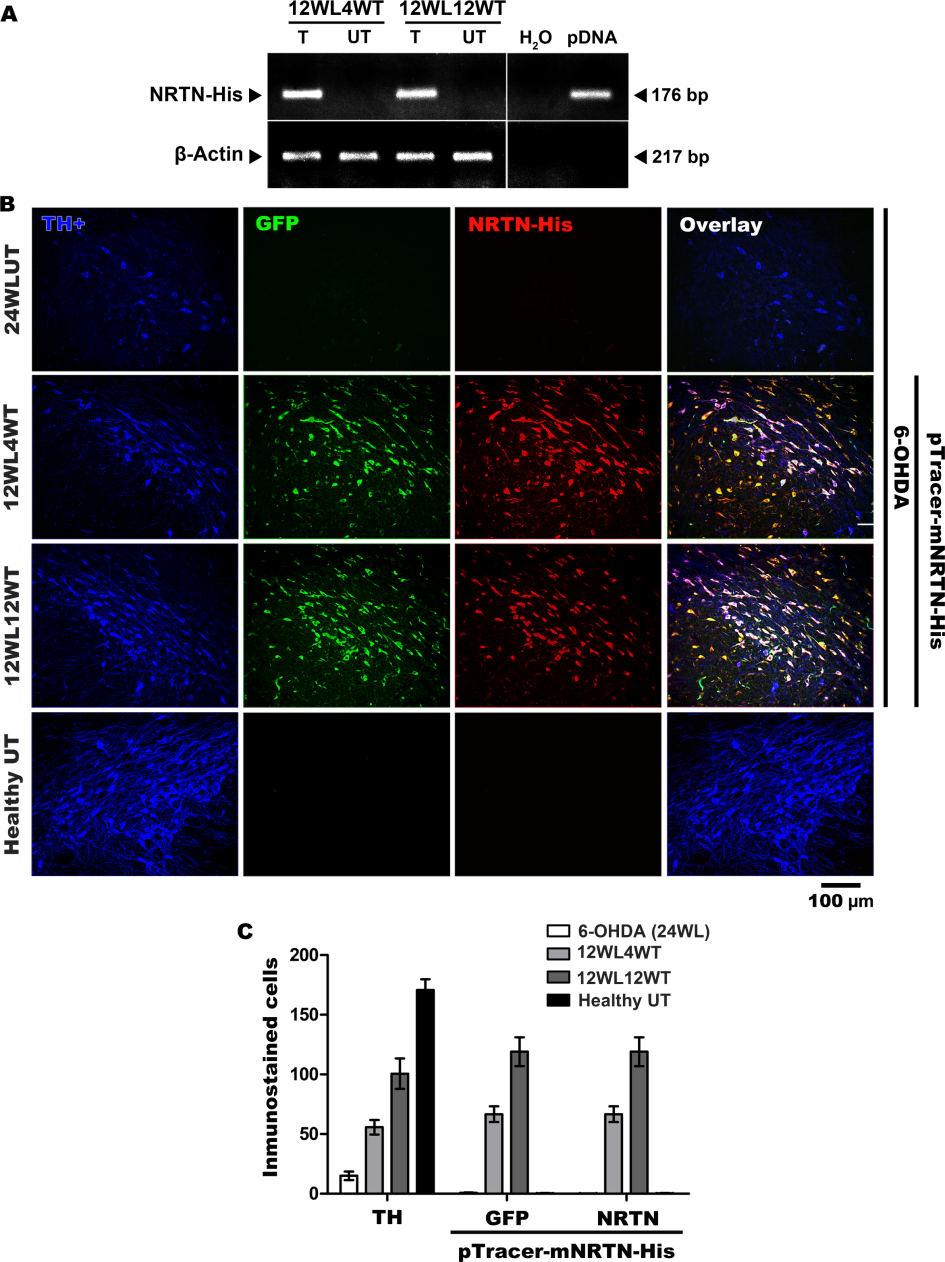
NRTN-His expression in the substantia nigra of rats with chronic 6-OHDA lesion. **A.** Representative agarose gel showing the amplicons for NRTN-His and β-actin after a RT-PCR assay. Internal controls in the absence (H_2_O) and presence of pTracer-mNRTN-His (pDNA). **B.** Representative micrographs of triple immunofluorescence assays against TH, the rate limiting enzyme in DA synthesis [53], green fluorescent protein (GFP) and His epitope (NRTN-His). **C.** Number of immunostained transfected cells. Intranigral transfection of the plasmid pTracer-mNRTN-His, which codes for NRTN-His and GFP, was made at week 12 after lesion. 12WL4WT = 12 weeks after lesion and 4 weeks after transfection. T = transfected side. UT = Untransfected side of the same rat. 12WL12WT = 12 weeks after lesion and 12 weeks after transfection. The scale bar is common for all micrographs.

NRTN-His mRNA was present in the substantia nigra of rats with chronic 6-OHDA lesion at months 1 and 3 after transfection of the plasmid pTracer-mNRTN-His, but absent in the untransfected contralateral substantia nigra (Fig 1A). The translated NRTN-His protein, as quantified by ELISA, was also present both in the substantia nigra and in the striatum, and protein levels in the substantia nigra were approximately 1.5-fold greater than those in the striatum when compared at the same time after transfection (Table 1). In the absence of transfection or in pGreenLantern-1 plasmid transfection, NRTN-His protein levels were undetected in those the nuclei studied (Table 1). The triple immunofluorescence assays showed the colocation of NRTN-His, GFP and TH in nigral cells after the transfection of pTracer-mNRTN-His, which codes for NRTN-His and GFP (Fig 1B). No immunoreactivity to NRTN-His and GFP was seen in untransfected rats (Fig 1B). The quantification studies showed an increase in the transgenic expression when recuperated the number of dopaminergic neurons (Fig 1C). These results demonstrate that the NTS-polyplex NPs system is able to transfect the residual dopaminergic neurons in the chronic model of 6-OHDA.

**Table 1:** NRTN-His levels measured with ELISA.

	NRTN-His (ng/mg protein)	
	Substantia nigra	Striatum
6-OHDA 24WL UT	ND	ND
12WL4WNRTN	8.866 ± 1.962 ^§^	4.566 ± 0.946
12WL12WNRTN	23.550 ± 4.125 *^,^^§^	16.274 ± 1.997 *
6-OHDA GFP	ND	ND
HEALTHY	ND	ND

6-OHDA 24WL UT = untransfected rats with 24 weeks of 6-OHDA lesion. 12WL4WNRTN = 12 weeks after lesion and 4 weeks after transfection of pTracer-mNRTN-His. 12WL12WNRTN = 12 weeks after lesion and 12 weeks after transfection of pTracer-mNRTN-His. 6-OHDA GFP = 12 weeks after lesion and 12 weeks after transfection of pGreenLantern-1. ND = not detectable. All values are the mean ± SEM (*n* = 3 independent rats for each experimental condition).

* *P* < 0.05 when compared with 12WL4WNRTN in the same nucleus.

§ *P* < 0.05 when compared with the striatum at the same time after transfection.

One-way ANOVA and Tukey post-test.

### Restoration of the nigrostriatal dopaminergic system

**Fig 2:**
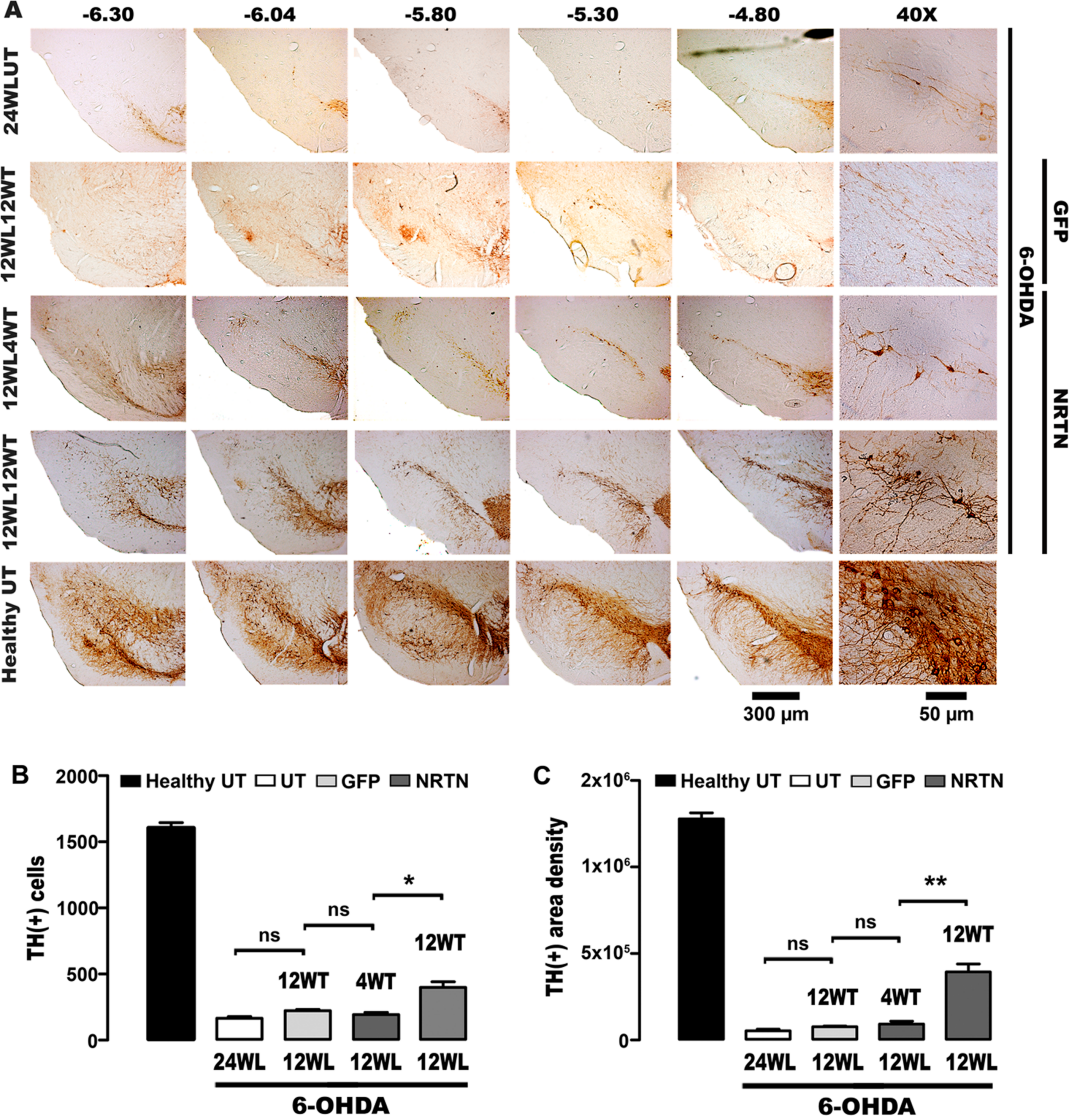
Neurotrophic effect of NRTN gene transfection on TH(+) cells and neurites of the substantia nigra with chronic 6-OHDA lesion. **A.** Representative micrographs of TH immunohistochemistry. Headings = Anterior-posterior coordinates of Paxinos and Watson Rat Atlas [54]. 24WLUT = Untransfected rats with 24 weeks of 6-OHDA lesion. 12WL4WT = 12 weeks after lesion and 4 weeks after transfection, 12WL12WT = 12 weeks after lesion and 12 weeks after transfection. The scale bar of 300 μm is common for slices at different levels and of 50 μm for details. **B.** TH(+) neuron counting. **C.** Densitometry of TH(+) relative area. 4WT and 12 WT = 4 and 12 weeks after transfection. 24WL = untransfected rats with 24 weeks of lesion. 12WL = 12 weeks after lesion. The transfections of pGreenLantern-1 (GFP) and pTracer-mNRTN-His (NRTN) plasmids were made at week 12 after lesion. All values are the mean ± SEM (*n* = 3 independent rats for each experimental condition). One-way ANOVA and Newman-Keuls post-test. * *P* < 0.001, ** *P* < 0.0001. ns = no statistical significance, *P* > 0.05.

**Fig 3:**
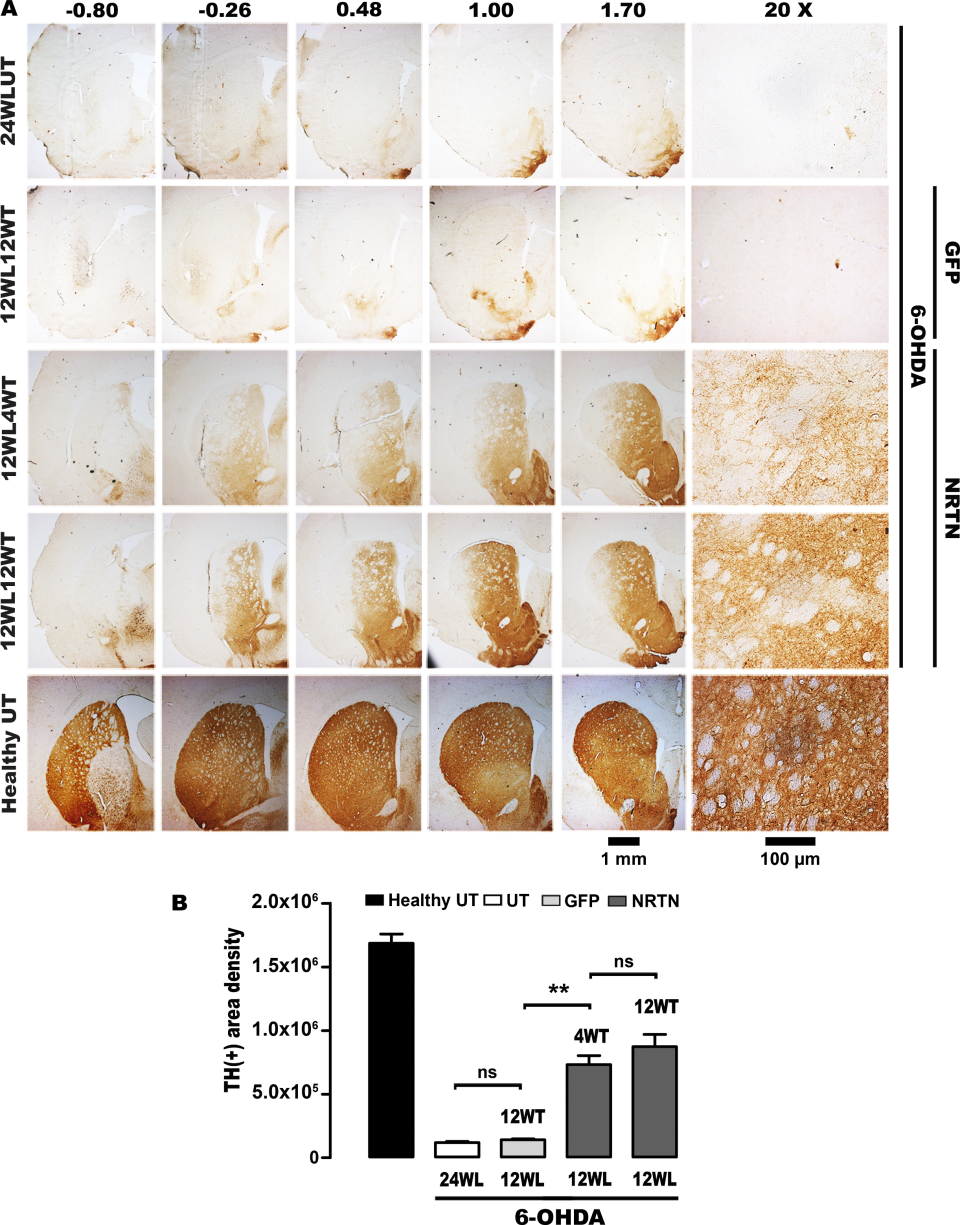
NRTN gene transfection increases TH(+) fibers in the striatum of rats with chronic 6-OHDA lesion. **A.** Representative micrographs of TH immunohistochemistry. Headings = Anterior-posterior coordinates of Paxinos and Watson Rat Atlas [54]. 24WLUT = Untransfected rats with 24 weeks of 6-OHDA lesion. 12WL4WT = 12 weeks after lesion and 4 weeks after transfection, 12WL12WT = 12 weeks after lesion and 12 weeks after transfection. The scale bar of 1 mm is for slices at different levels and of 100 μm for details. **B.** Densitometry of TH(+) relative area. 4WT and 12 WT = 4 and 12 weeks after transfection. 24WL = untransfected rats with 24 weeks of lesion. 12WL = 12 weeks after lesion. The transfections of pGreenLantern-1 (GFP) and pTracer-mNRTN-His (NRTN) plasmids were made at week 12 after lesion. One-way ANOVA and Newman-Keuls post-test. * *P* < 0.001, ** *P* < 0.0001. ns = no statistical significance, *P* > 0.05.

At the end of the study, the loss of TH(+) cells was 88% and the decrease of TH(+) fibers was 94% in the substantia nigra with 6-OHDA lesion (Fig 2), whereas the decrease of TH(+) fibers in the striatum with lesion was 92% (Fig 3). No neurotrophic effect on the nigrostriatal system was seen in rats transfected with the plasmid pGreenLantern-1 (Figs 2 and 3). In contrast, the transfection of pTracer-mNRTN-His plasmid increased the number of TH(+) cells by 157%, as compared with the untransfected substantia nigra with lesion (Fig 2B) at the end of the study. At the time, TH-immunoreactivity density was 7-fold higher than that of the untransfected substantia nigra with lesion (Fig 2C). The recovery reached 28 ± 2% of TH(+) cell population and 32 ± 2% of the neuropil density when compared with the healthy condition at the end of the study (Fig 2). The neurotrophic effect was more pronounced in the striatum (Fig 3). The increase in the density of TH(+) fibers was statistically significant at the first month after transfection when compared with the striatum with lesion, reaching 52 ± 3% of density of the healthy condition at the end of the study (Fig 3B). The recovery of TH(+) cells and their branching in the substantia nigra (Fig 2A) and of TH(+) fibers in the striatum (Fig 3A) occurred along the whole nucleus.

**Fig 4:**
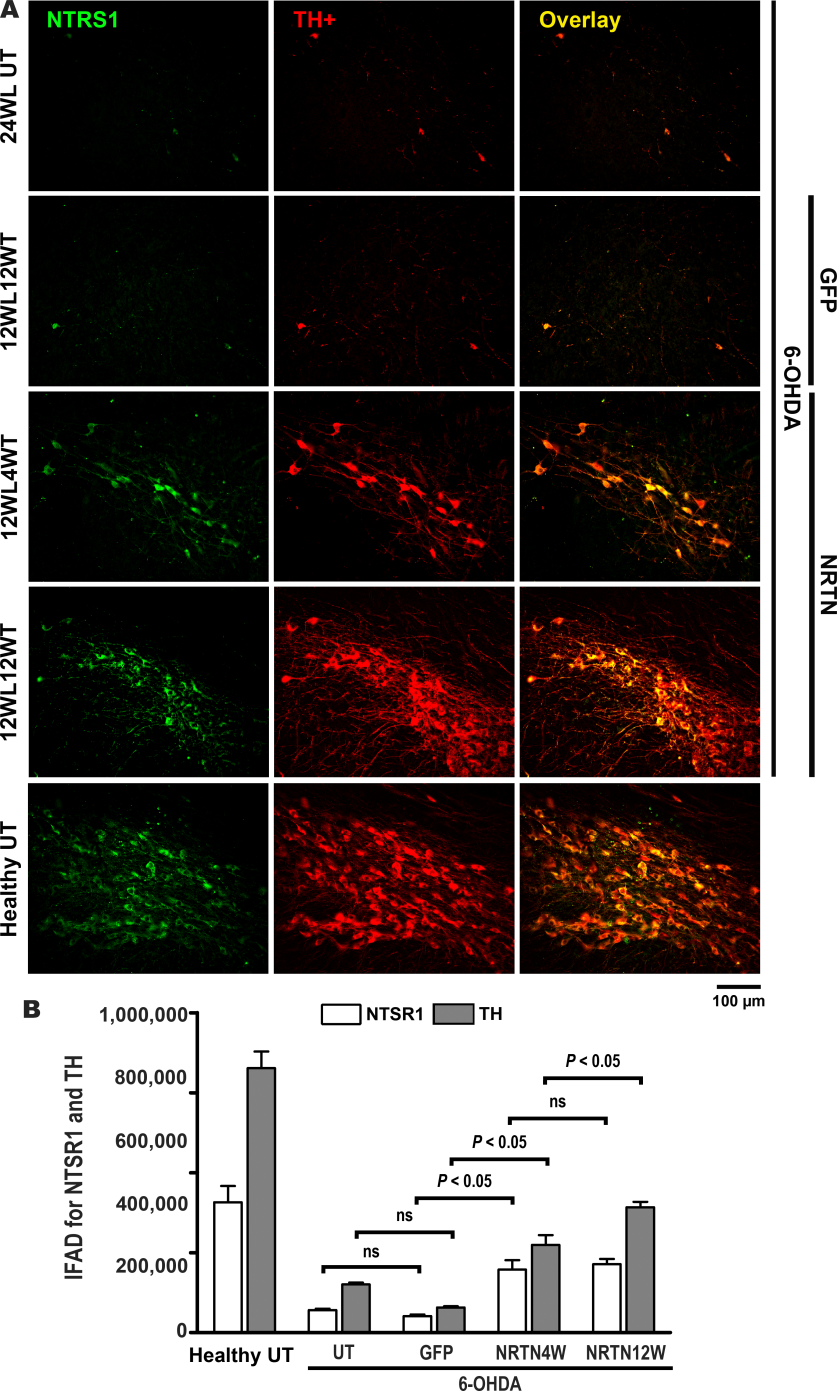
NRTN gene transfection increases NTSR1 levels in the substantia nigra of rats with chronic 6-OHDA lesion. **A.** Representative micrographs of double immunofluorescence staining against NTSR1 and TH. 24WLUT = Untransfected rats with 24 weeks of 6-OHDA lesion. 12WL4WT = 12 weeks after lesion and 4 weeks after transfection, 12WL12WT = 12 weeks after lesion and 12 weeks after transfection. The scale is common for all micrographs. **B.** immunofluorescence area density (IFAD) for NTSR1 and TH was determined using ImageJ software v.1.46r (National Institutes of Health; Bethesda, MD). NRTN4WT and NRTN412WT = 4 and 12 weeks after transfection. UT = untransfected rats. The transfections of pGreenLantern-1 (GFP) and pTracer-mNRTN-His (NRTN) plasmids were made at week 12 after lesion. All values are the mean ± SEM (*n* = 3 independent rats for each experimental condition). Two-way ANOVA and Bonferroni post-test. ns = no statistical significance.

**Fig 5:**
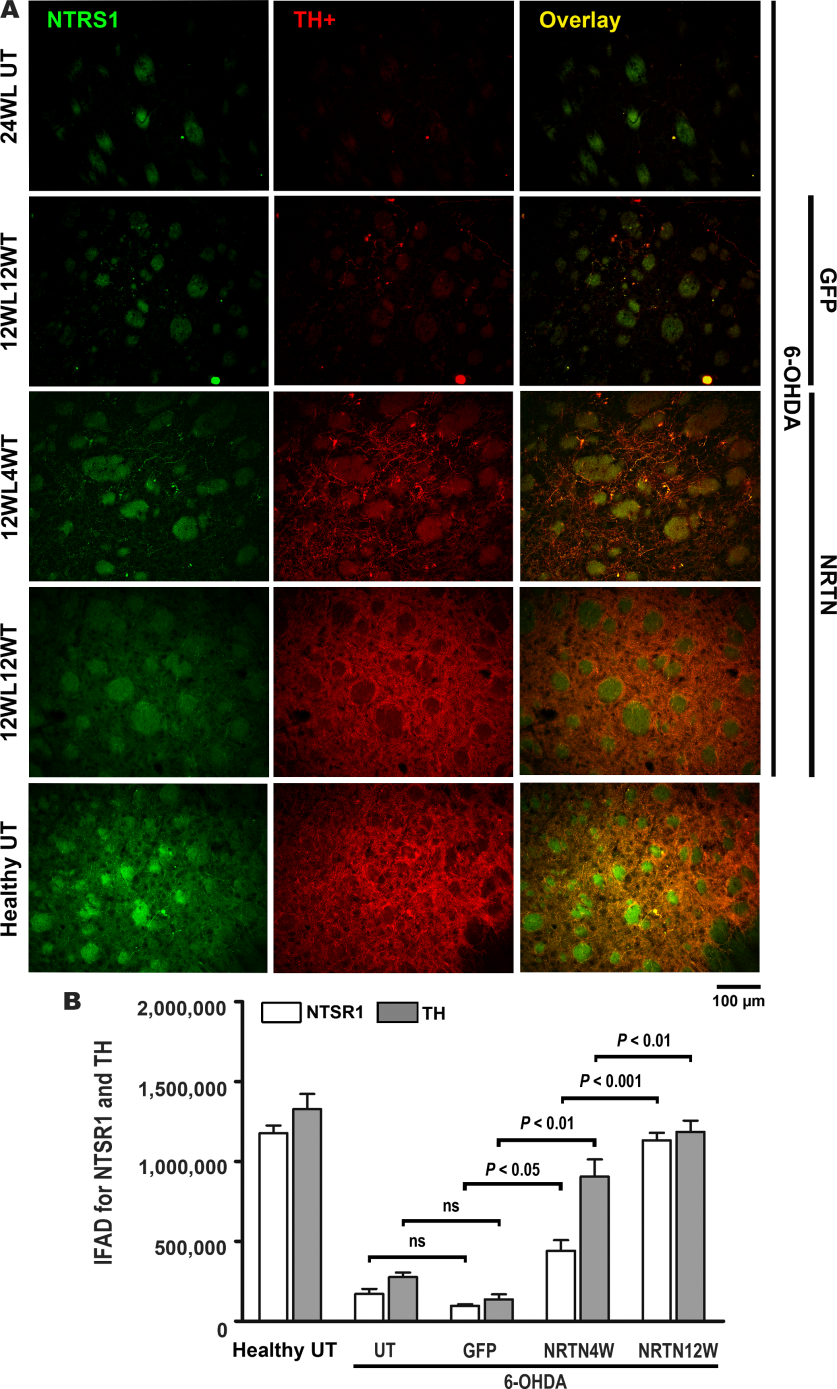
NRTN gene transfection increases NTRS1 levels in the striatum of rats with chronic 6-OHDA lesion. **A.** Representative micrographs of double immunofluorescence staining against NTSR1 and TH. 24WLUT = Untransfected rats with 24 weeks of 6-OHDA lesion. 12WL4WT = 12 weeks after lesion and 4 weeks after transfection, 12WL12WT = 12 weeks after lesion and 12 weeks after transfection. The scale is common for all micrographs. B. immunofluorescence area density (IFAD) for NTSR1 and TH was determined using ImageJ software v.1.46r (National Institutes of Health; Bethesda, MD). NRTN4WT and NRTN412WT = 4 and 12 weeks after transfection. UT = untransfected rats. The transfections of pGreenLantern-1 (GFP) and pTracer-mNRTN-His (NRTN) plasmids were made at week 12 after lesion. All values are the mean ± SEM (n = 3 independent rats for each experimental condition). Two-way ANOVA and Bonferroni post-test. ns = no statistical significance.

Although NTSR1 is not a marker of dopaminergic phenotype, this receptor is present in nigral dopaminergic neurons at high density [33], decreases in the substantia nigra of PD patients [55] and of 6-OHDA parkinsonian rats [28], and plays an important role in the modulation of nigrostriatal dopaminergic neurotransmission [56]. Using double immunofluorescence analysis against NTSR1 and TH, we explored whether NRTN-His overexpression can also recover NTSR1 levels in the substantia nigra (Fig 4) and the striatum (Fig 5). The effect of 6-OHDA, pGreenLantern-1 plasmid transfection and pTracer-mNRTN-His plasmid transfection on IFAD for TH (Fig 4B and Fig 5B) agree with those on densitometry of TH relative area quantified in substantia nigra (Fig 2C) and striatum (Fig 3B) slices stained by immunohistochemistry. IFAD for NTSR1 was lower than that for TH in both nuclei studied of the healthy untransfected controls (Figs 4B and 5B), suggesting that NTSR1 protein levels are normally lower than TH protein levels. The transfection of pGreenLantern-1 plasmid did not modify the reduced IFAD for NTSR1 in the substantia nigra (Fig 4B) and striatum (Fig 5B) of rats with 6-OHDA lesion. In contrast, the transfection of pTracer-mNRTN-His plasmid caused a significant increase in IFAD for NTSR1 in the substantia nigra (Fig 4B) and striatum (Fig 5B) of rats with 6-OHDA lesion as compared with the respective controls with lesion. NTSR1 increase was higher in the striatum (Fig 5B) than in the substantia nigra (Fig 4B) at the two times studied (Fig 4B) similar to the quantification of immunohistochemistry staining (Fig 2C and Fig 3B). Details of organization and thickening of nigrostriatal terminals at the entrance of the medial forebrain bundle into the striatum at months 1 and 3 after transfection are shown in S1 Fig. These results demonstrate that the neurotrophic effect of NRTN-His overexpression is exerted not only on dopaminergic phenotype (TH), but also on a different protein (NTSR1) that modulates the activity of nigral dopaminergic neurons [56].

### Restoration of neuronal cytoskeleton

**Fig 6:**
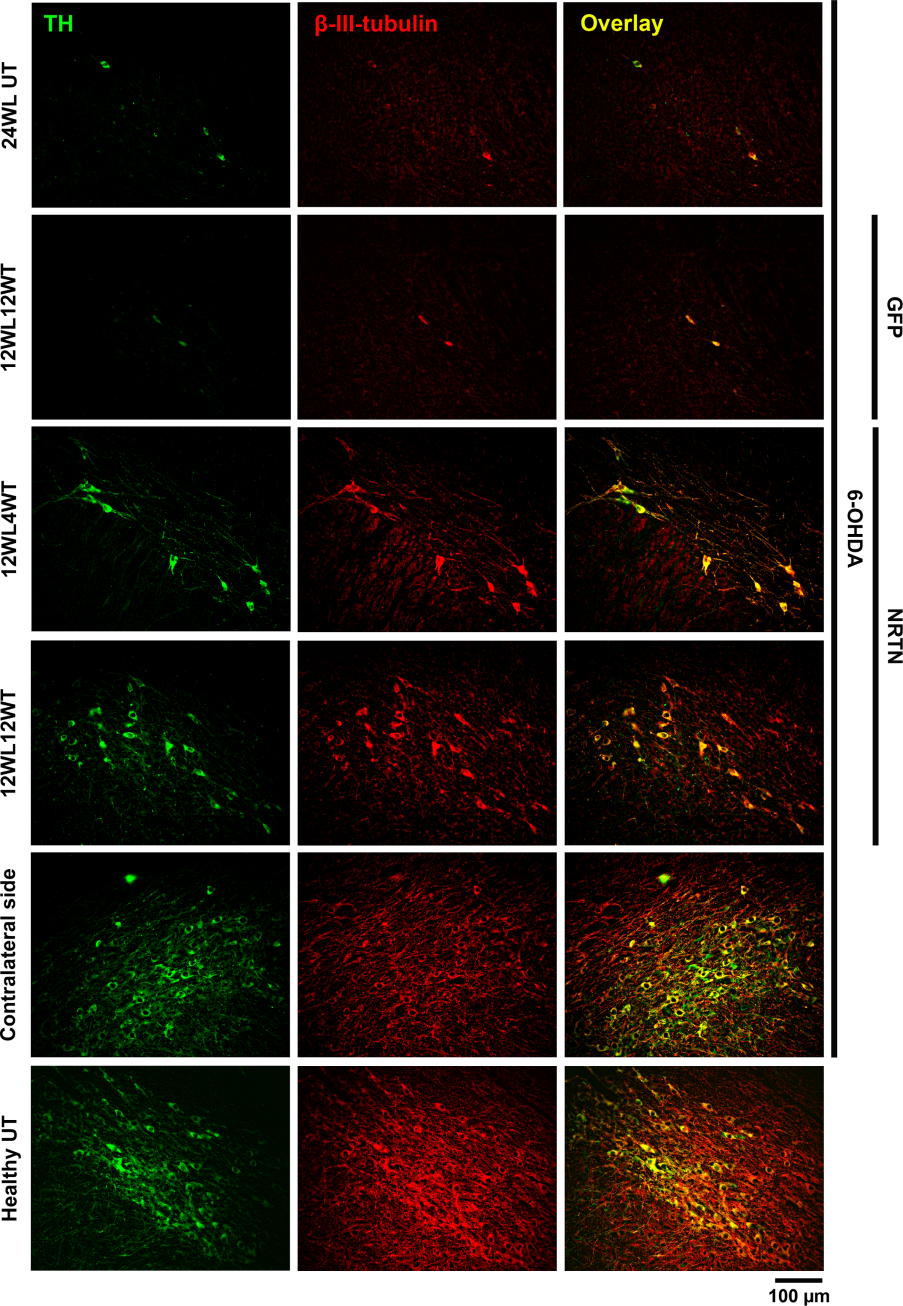
Neuronal cytoskeleton restoration after NRTN-His NPs transfection. Representative micrographs of double immunofluorescence to TH and β-III-tubulin. 24WLUT = Untransfected rats with 24 weeks of 6-OHDA lesion. 12WL4WT = 12 weeks after lesion and 4 weeks after transfection, 12WL12WT = 12 weeks after lesion and 12 weeks after transfection. The transfections of pGreenLantern-1 (GFP) and pTracer-mNRTN-His (NRTN) plasmids were made at week 12 after lesion. The scale bar is common for all micrographs.

The control substantia nigra without lesion showed that the cells displayed immunoreactivity to TH and β-III-tubulin (Fig 6), a neuron-specific cytoskeletal marker [57]. A significant loss of the double immunoreactivity occurred in the substantia nigra of untransfected animals with chronic 6-OHDA lesion (Fig 6), thus suggesting the disappearance of neuronal cytoskeleton because of dopaminergic cell death [26]. The transfection of pGreenLantern-1 plasmid did not alter the 6-OHDA-induced loss of TH and β-III-tubulin immunoreactivity (Fig 6). In contrast, the transfection of pTracer-mNRTN-His plasmid caused an increasing recovery of cells with double immunoreactivity to TH and β-III-tubulin over time (Fig 6), suggesting the restoration of neuronal cytoskeleton in the rescued dopaminergic neurons.

### Recovery of dopamine content in the nigrostriatal dopaminergic system

**Fig 7:**
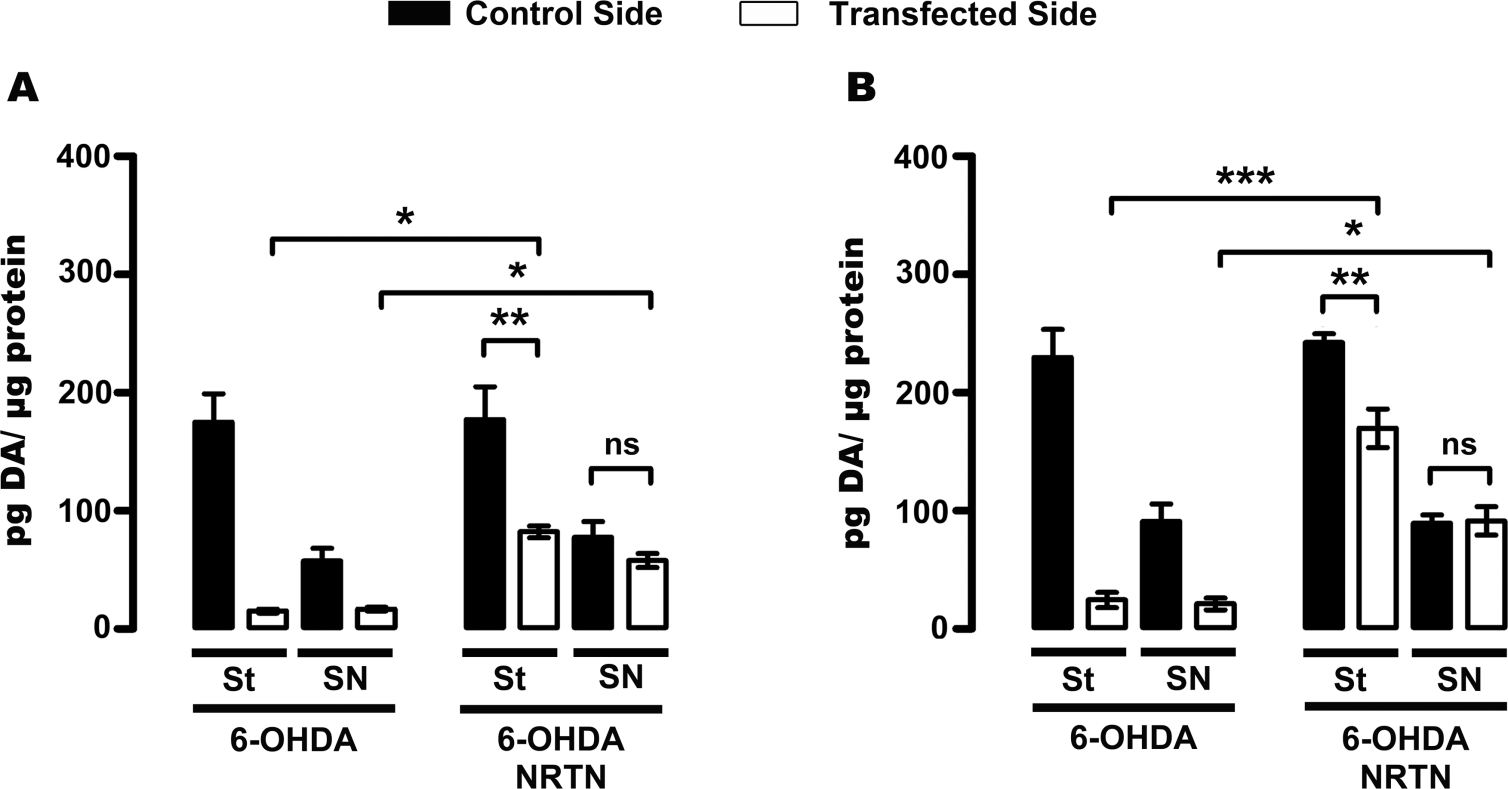
NRTN gene transfection recovers dopamine levels in the substantia nigra (SN) and striatum (St) of rats with chronic 6-OHDA lesion. HPLC measurements of dopamine levels were made at weeks 4 (**A**) and 12 (**B**) after pTracer-mNRTN-His (NRTN) transfection made at week 12 after the lesion. All values represent mean ± SEM (*n* = 4 independent rat in each experimental condition). One-way ANOVA and Newman-Keuls post-test. *P < 0.05, **P < 0.01, ***P < 0.001. ns = non statistical significance, *P* > 0.05.

A profound loss of dopamine levels in both the striatum (93 ± 1%) and substantia nigra (75 ± 2%) occurred at week 16 after intrastriatal 6-OHDA injection when compared with the levels of the healthy, contralateral side (Fig 7A); those decrements remained until week 24 after lesion, the end of the study (Fig 7B). The rats with one month of transfection with the plasmid pTracer-mNRTN-His showed 5-fold increase in dopamine levels in the striatum and 4-fold increase in the substantia nigra when compared with their control sides (Fig 7A). At the end of study, the recovery of dopamine levels was 70 ± 4% in the striatum and was complete in the substantia nigra in comparison with the control side (Fig 7B).

### Restoration of dendritic spines

**Fig 8:**
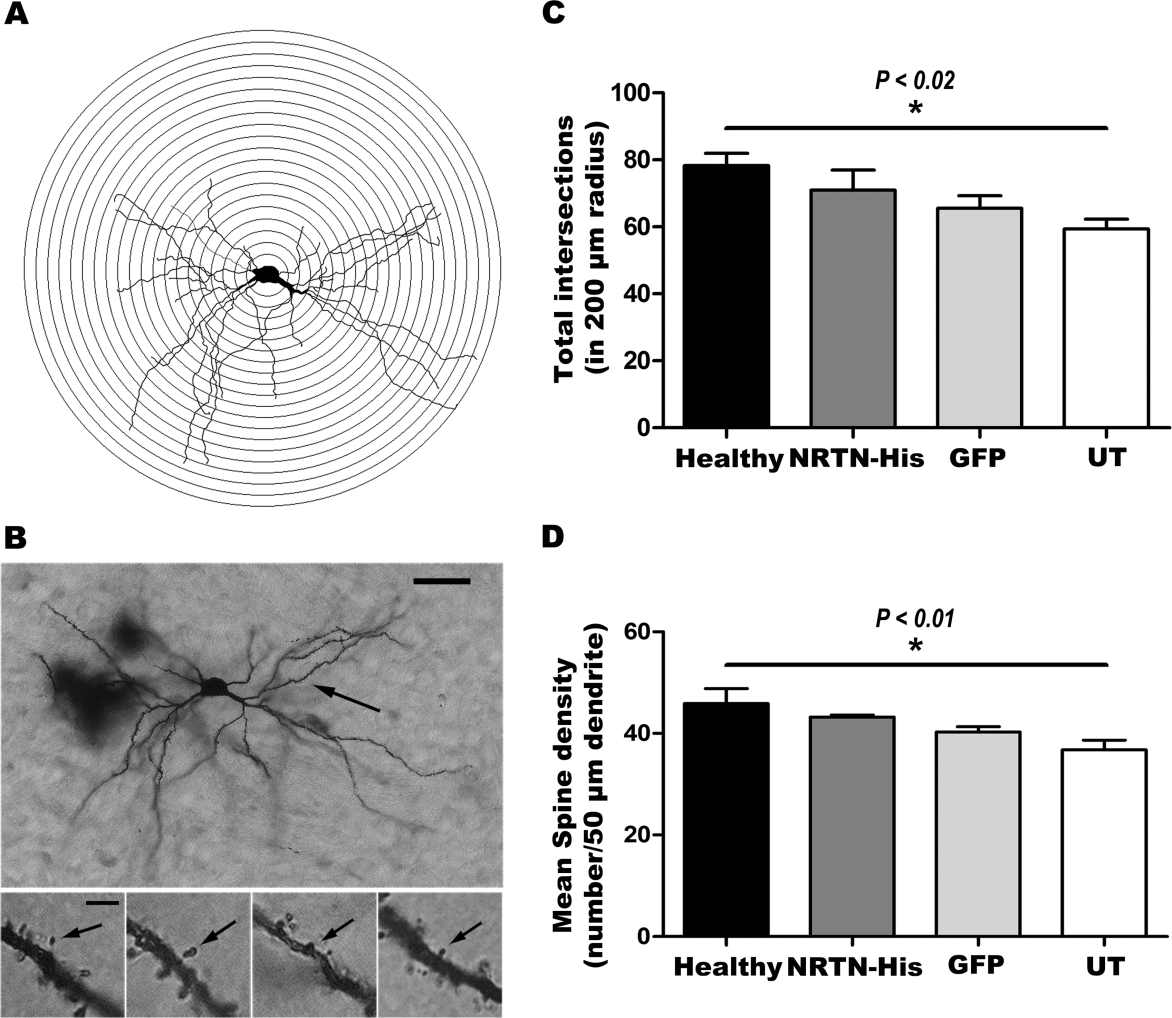
NRTN gene transfection increase spine density and total number of intersections in MSN. **A**. Sholl diagram for the morphological study of dendrites and dendritic spines. **B**. Representative micrograph of a medium spiny neuron (MSN) of a healthy striatum. The arrow shows the 50 μm segment (primary dendrite) where dendritic spines were analyzed. Upper calibration bar = 25 μm. The small panels show different types of spines (thin, mushroom, stubby and wide, from left to right) indicated by the arrow. Calibration bar = 5 μm. The transfections of pGreenLantern-1 (GFP) and pTracer-mNRTN-His (NRTN) plasmids were made at week 12 after lesion and the Sholl analysis was performed at the end of the study (12 weeks after transfection or 24 weeks after lesion). UT = untransfected rats with lesion. **C.** Sholl analysis of total number of intersections along dendritic trees in MSNs at all distances in 200-μm radius from the soma. **D.** Plot of mean spine density analyzed per 50-μm primary dendrite from cell body (proximal segment, upper arrow in **B**) from six neurons per rat. All values represent the mean ± SEM (*n* = 6 independent rats in each experimental condition). Statistical analysis (**C** and **D**) was performed using one-way ANOVA and Tukey *post-hoc* test.

As reported previously [25], 6-OHDA lesion significantly decreased the branching of dendrites and the density of dendritic spines on the striatal MSNs in comparison with the healthy condition (Fig 8). Of the four different types of spines studied, 6-OHDA lesion only decreased the density of the thin spines significantly (Table 2). pGreenLantern-1 transfection caused a modest increase of the dendritic arborization and in the length and density of dendritic spines (Fig 8), mainly of thin spines of MSNs (Table 2), as compared with the lesion condition in untransfected animals. The maximum increase in those variables of dendritic arborization and dendritic spines was significantly caused by the transfection of pTracer-mNRTN-His, although the recovery did not reach the values of the healthy condition (Fig 8 and Table 2).

**Table 2:** Proportional density of the different types of spines in medium spiny neurons from the rats of the four groups studied.

	Group			
Spine Type	Healthy	NRTN-His	GFP	UT
**Thin**	29.9 ± 1.7	24.7 ± 0.4 ^a^	24.9 ± 0.9 ^a^	22.4 ± 1.2 ^a^
**Mushroom**	10.0 ± 0.5	11.4 ± 0.3 ^b^ ^c^	8.4 ± 0.4	8.3 ± 0.5
**Stubby**	2.4 ± 0.3	2.7 ± 0.2	2.9 ± 0.1	3.0 ± 0.1
**Wide**	2.0 ± 0.3	2.5 ± 0	1.7 ± 0.3	1.7 ± 0.2

Plasmids pGreenLantern-1 (GFP) or pTracer-mNRTN-His (NRTN) were transfected in rats with 12 weeks of lesion. UT = untransfected rats with lesion. All values represent the Mean ± SEM (*n* = 6 independent rats in each experimental condition). *P* < 0.05

a: vs. Healthy

b: vs. Lesion

c: vs. GFP.

One-way ANOVA and Bonferroni *post-hoc* test.

### Decrease of drug-induced and spontaneous motor impairments

**Fig 9:**
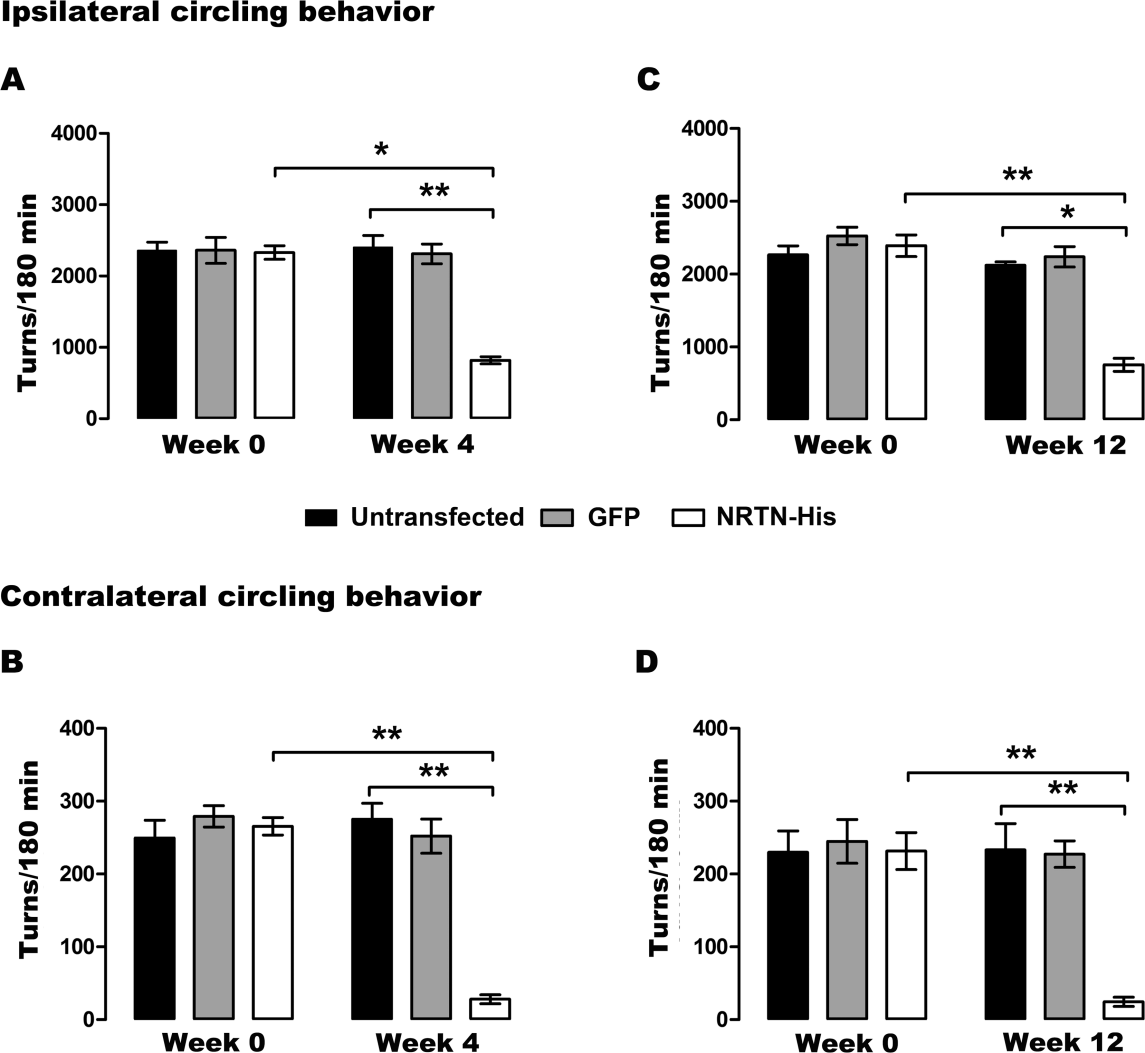
Effect of NRTN gene transfection on circling behavior of rats with chronic 6-OHDA lesion. Ipsilateral circling behavior (**A and C**) was activated by methamphetamine (8 mg/kg; i.p.), and contralateral circling behavior (**B and D**) was activated by apomorphine (0.5 mg/kg; i.p.) at weeks 0, 4 and 12 after transfection. The transfections of pGreenLantern-1 (GFP) and pTracer-mNRTN-His (NRTN) plasmids were made at week 12 after lesion (Week 0). All values represent the mean ± SEM (*n* = 4 independent rats in each experimental condition). One-way ANOVA and Newman-Keuls post-test. *P <0.0005, **P < 0.0001.

The circling behavior that was activated by methamphetamine in rats with 12 weeks of lesion (day 0 of transfection) ranged from 2263 to 2357 ipsilateral turns/180 min (Fig 9A). Whereas, the apomorphine-activated circling behavior ranged from 229 to 249 contralateral turns/40 min (Fig 9B). Those circling behaviors remained constant until the end of the study (Fig 9C and 9D). pGreenLantern-1 transfection did not alter dopamine agonist-activated circling behaviors (Fig 9). In contrast, pTracer-mNRTN-His transfection decreased dopamine agonist-activated circling behavior 65 ± 2% (methamphetamine) and 89 ± 1% (apomorphine) at week 4 after transfection (Fig 9A and 9B), and 73 ± 2% (methamphetamine) and 89 ± 1% (apomorphine) at week 12 after transfection (Fig 9C and 9D). These results strongly suggest that the expression of NRTN-His in the lesion side significantly restored the functional symmetry of the nigrostriatal system.

**Fig 10:**
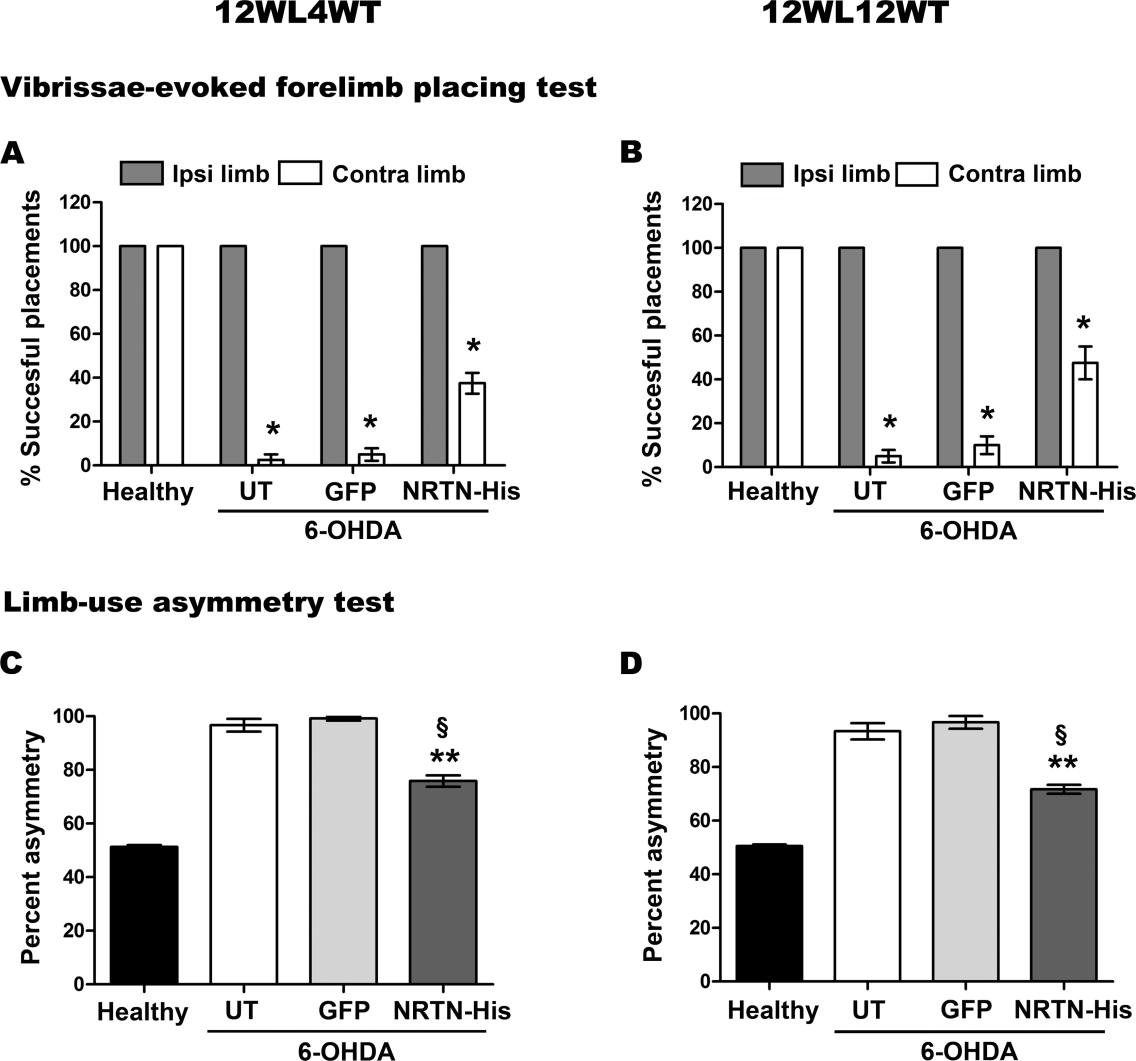
Vibrissae-evoked forelimb placed test and cylinder test. The transfections of pGreenLantern-1 (GFP) and pTracer-mNRTN-His (NRTN) plasmids were made at week 12 after lesion. UT = untransfected rats. The tests were performed at week 4 (12WL4WT) and week 12 (12WL12WT) after transfection. All values represent the mean ± SEM (*n* = 4 independent rats in each experimental condition). *P < 0.001 when compared the values of the contralateral limb (Contra limb) with those of the ipsilateral limb (Ipsi limb), **P < 0.001 when compared NRTN-His vs UT and ^§^*P* < 0.001, NRTN-His vs GFP using two-way ANOVA. The *post-hoc* test was Newman-Keuls analysis.

The 6-OHDA-induced asymmetry could also be revealed by the vibrissae forelimb placing test (Fig 10A and 10B) and limb-use asymmetry test (Fig 10C and 10D). In both cases, the motor impairment occurred in the forelimb of the contralateral side to the lesion and remained until the end of the study (Fig 10A–10D). pGreenLantern-1 transfection did not modify either the forelimb responses to tactile stimuli (Fig 10A and 10B) or the spontaneous use of the forelimb (Fig 10C and 10D) at week 4 and week 12 after transfection. In contrast, pTracer-mNRTN-His transfection significantly improved the response to tactile stimuli and the placements of the affected forelimb at weeks 4 and 12 after transfection, as compared with the untransfected animals and those transfected with the plasmid pGreenLantern-1 (Fig 10A–10D). However, the improvement induced by the pTracer-mNRTN-His transfection in the drug-activated behavior and spontaneous behavior did not reach the reference values of the healthy condition (Figs 9 and 10).

## Discussion

Our results show, for the first time, that NRTN overexpressed in dopamine neurons of the substantia nigra promotes structural plasticity of the nigrostriatal system in the rat with chronic 6-OHDA lesion. The major changes in presynaptic structures were preservation of an increased number of dopamine neurons in the substantia nigra and sprouting of nigral neurites and striatal terminals as well as a significant recovery of NTSR1, whereas the postsynaptic changes consisted of increase in dendritic spines of striatal neurons, when compared with the lesion conditions. The level of restoration achieved in the study period enabled the re-establishment of biochemical and functional activity of dopaminergic nigrostriatal pathway from week 4 post-transfection. The ability of NRTN to stimulate axonal elongation and dendritic branching in cell cultures and in experimental animals [18] can account for the sprouting of dopaminergic fibers in the substantia nigra and in the striatum. However, the increased number of dopaminergic neurons cannot be convincingly explained by an effect of NRTN on survival of mesencephalic neurons because this issue is still controversial [7, 18] and experimental evidence on repopulation of dopamine neurons in the adult brain has not yet been reported. The controversy might have resulted from differences in the kind of neural population, the lesion model, duration of the study and the transfection method.

For this study, we used the lesion model of a single injection of 6-OHDA (20 μg) in the divergence site of the medial forebrain bundle in the striatum that activates apoptosis in nigral dopaminergic neurons within a limited time course [26]. In this model, the apoptotic process and cytoskeleton disorganization last 4 weeks, and thereafter 10 to 20% of TH(+) cells remain much longer than 8 weeks after the lesion [26]. Our results in untransfected rats with chronic lesion confirm the presynaptic impairment of dopaminergic nigrostriatal pathway as longer as 24 weeks of lesion [26]. As expected, the increasing concentration of NRTN-His over time was accompanied by an increasing appearance of neuronal cytoskeleton and a robust branching of TH(+) fibers in the substantia nigra. Furthermore, the increased cytoskeleton immunoreactivity in the neuron body collocated with an increased number of TH(+) neurons, thus suggesting recovery of dopaminergic neuron population. Such a recovery might not be the result from an antiapoptotic effect, because this event ends 4 weeks after the 6-OHDA lesion [26]. Then, a possibility would be that the increased number of dopaminergic neurons might result from differentiation of progenitor cells of the substantia nigra that have the potential of generating new neurons in rodents [58–60]. However, others studies have found a significant increase in numbers of newborn NG2-positive and GFAP-positive cells in the substantia nigra of rodents [61–63] instead of new dopaminergic neurons [28], even after the treatment with GDNF [61] or BDNF [28, 62]. These studies suggest that gliogenic stimuli inducing significant micro-environmental changes in the adult substantia nigra might improve motor behavior in the 6-OHDA lesion rats. Other possibilities are that NRTN might have induced the dopaminergic phenotype in TH(-) neurons residents in the substantia nigra [64] or might rescue the dopaminergic phenotype [61, 65] in that population of surviving neurons that were not quantified in the 6-OHDA lesion model used for this study [26]. Further investigation is necessary to gain insight into the exact mechanism by which NRTN-His elicits repopulation of nigral dopaminergic neurons in the chronic lesion 6-OHDA model in the rat.

The levels of NRTN-His in the substantia nigra and the striatum after NTS-polyplex NPs nigral gene delivery were in the nanogram range similar to human (h)NRTN levels following intrastriatal administration of a serotype 2 adeno-associated (AAV2-NRTN) viral vector encoding hNRTN and increased over time [66]. The NRTN-His levels were also similar to those achieved after direct infusion of NRTN (10 μg) or GDNF (15 μg) into the rat brain [67]. The levels in the nanogram range (from 4.566 to 60 ng, in total) achieved by those three independent delivery techniques showed bioactivity of NRTN as shown by our results and others [66, 67]. Interestingly, NRTN-His levels in the striatum corresponded to almost 40% of those in the substantia nigra after nigral gene delivery. NRTN is well known for its poor extracellular diffusion caused by its strong binding to extracellular matrix and cell surface heparan sulfate proteoglycans [68, 69]. Therefore, NRTN diffusion cannot explain the high levels of NRTN-His in the striatum and the His-tag is also unlikely to modify the diffusion rate of NRTN. We propose that the selectivity of NTS-polyplex NPs to transfect dopaminergic neurons accounts for the high striatal levels of NRTN-His. Accordingly, the overexpression of NRTN-His in the dopaminergic neurons could favor the axonal transport of NRTS-His to the striatum that could also be potentiated by the outgrowth of the nigrostriatal terminals and the restoration of neuronal cytoskeleton as shown herein. It is well known that neuronal cytoskeleton plays a key role in the anterograde axonal transport of neurotrophic factors [70–72].

We found that 6-OHDA caused a significant loss of dopamine content in the striatum that was accompanied by a decreased number and altered morphology of dendritic spines of MSNs. These results confirm previous results in the 6-OHDA rat model and gives further support to the neurotrophic role of dopamine in the striatum during adulthood [25, 30]. Then, dopamine and NRTN-His in the striatum might have caused the recovery of dendritic spines of MSNs. However, the restitution of dendritic spines was not as complete as that caused by the combined treatment of a D3 agonist and BDNF transfection [30]. While, NRTN can exert neuroprotection in striatal neurons [73], it is possible that the co-transfection of BDNF gene can be required to achieve a complete recovery of striatal dendritic spines of MSNs [30, 74]. In addition, a previous work has shown that the maintenance of dendritic spines of MSNs is essential for the complete and enduring recovery of the physiological motor behavior [30]. Accordingly, a significant increase of spontaneous motor behavior and decrease of drug-activated circling behavior was only present in rats transfected with NRTN-His till the end of the study (3 months after transfection). The decrease of apomorphine-induced circling behavior supports the hypersensitivity reduction of postsynaptic dopamine receptors possibly caused by their continuous activation by dopamine levels, which were high in the striatum of rats transfected with NRTN-His gene. However, the decrease did not reach the normal basal values as evaluated in all behavioral tests in agreement with the partial recovery of medium spiny dendrites shown by this study. In addition, the recovery of NTSR1 levels in TH(+) neurons and striatal terminals was also partial. All these results together suggest that NRTN is lesser potent than GDNF or BDNF combined with a D3 agonist to restore the dopaminergic system in the 6-OHDA lesion model. This difference can be because NRTN would use GFRα1 [4, 5], Growth arrest specific 1 [75, 76], or independent-RET pathway receptors such as NCAM [77–79], integrin β-1 [80] and heparan sulfate proteoglycan syndecan-3 [68] for signaling instead of its high affinity natural GFRα2, which has not yet been convincingly demonstrated in nigral dopaminergic neurons. Another cause might be the poor diffusion and stability of NRTN because of its capacity to bind heparan sulfates in the extracellular matrix [2]. This is why, new variants of NRTN with high stability and wide diffusion capacity has recently been developed [2]. A final possibility might be that V5 and His tags on C-terminal could decrease NRTN bioactivity based on the finding that the C-terminus on GDNF is very close to the receptor-binding site according to the crystal structure of GDNF-GFRalpha1 complex [81].

NRTN gene transduction in the putamen of PD patients using AAV2-NRTN has been the first open-labeled clinical trial of restorative therapy [82]. Intraputaminal AAV2-NRTN-gene therapy showed clinical improvement in early stage PD [83] and its results of phase I clinical trials were positive [84]. Because of the limited, extracellular diffusion of NRTN, the relative clinical success might be attributed to the presence of a sufficient number of nigrostriatal fibers at early stage PD that might have enabled the retrograde transport of NRTN to the cell body in the substantia nigra where NRTN is needed to achieve a robust neurotrophic response [85]. This suggestion receives further support by our results showing that NRTN overexpression in dopaminergic neurons can stimulate neurite outgrowth in the nigrostriatal pathway and striatal dendritic spines in aging rats with chronic 6-OHDA lesion.

In conclusion, this work demonstrates that a single transfection of the plasmid pTracer-mNRTN-His into the dopaminergic neurons using NTS-polyplex NPs caused sustained NRTN-His levels in the substantia nigra and in the striatum that were accompanied with structural, biochemical and functional recovery of the chronically damaged dopaminergic nigrostriatal system. However, those improvements did not reach the healthy condition, suggesting that NRTN exerts lesser neurotrophic effects than GDNF or other combined treatments such as BDNF with D3 agonist. Our results open the possibility to test the new variants of NRTN or in combinations with other therapeutic strategies.

## Supporting information

S1 Fig Maturation of nigrostriatal terminals in the medial forebrain bundle arriving into the striatum after NRTN-His gene therapy. Details of nigrostriatal terminals with NTSR1 and TH double immunostaining (arrowheads) of rats with chronic 6-OHDA lesion at months 1 (12WL12WT) and 3 (12WL24WT) after transfection.
(TIF)
