# Supplementary figures and images for: Neurturin overexpression in dopaminergic neurons induces presynaptic and postsynaptic structural changes in rats with chronic 6-hydroxydopamine lesion
Source: PLoS One. 2017 Nov 27;12(11):e0188239. doi: 10.1371/journal.pone.0188239 (PMC5703459; doi:10.1371/journal.pone.0188239)

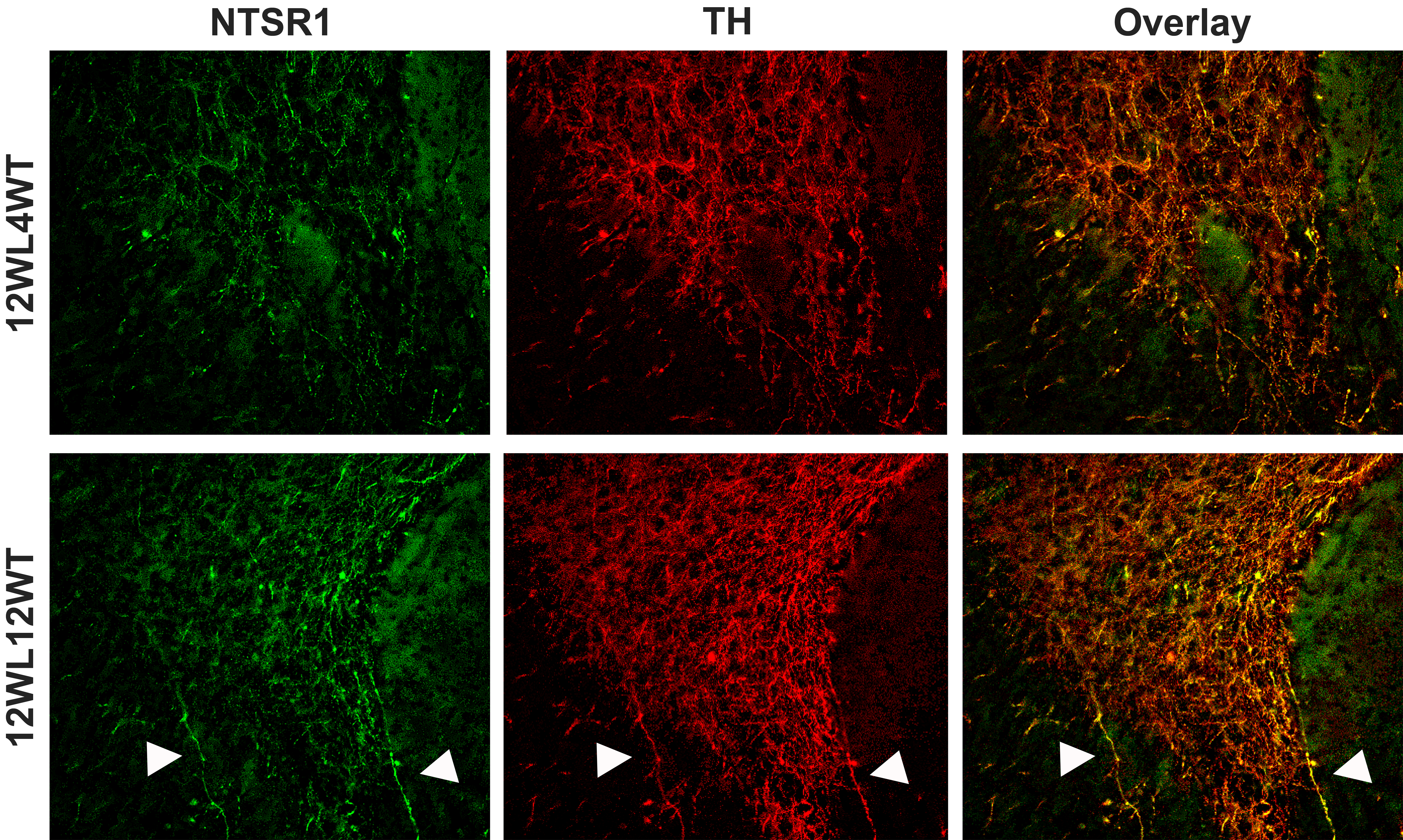

Supplement: S1 Fig — Details of nigrostriatal terminals with NTSR1 and TH double immunostaining (arrowheads) of rats with chronic 6-OHDA lesion at months 1 (12WL12WT) and 3 (12WL24WT) after transfection. (TIF) [file pone.0188239.s001.tif]
